# Supplementary material for: Efficient and reproducible generation of human iPSC-derived cardiomyocytes and cardiac organoids in stirred suspension systems
Source: Nat Commun. 2024 Jul 15;15:5929. doi: 10.1038/s41467-024-50224-0 (PMC11251028; doi:10.1038/s41467-024-50224-0)
Supplement: Supplementary file 1 — Supplementary Information [file 41467_2024_50224_MOESM1_ESM.pdf]

Supplementary Information

1. Figures S1-S10
2. Tables S1-S5
3. Extended Data 1-2
4. Movies S1-S11

Supplementary Figure 1

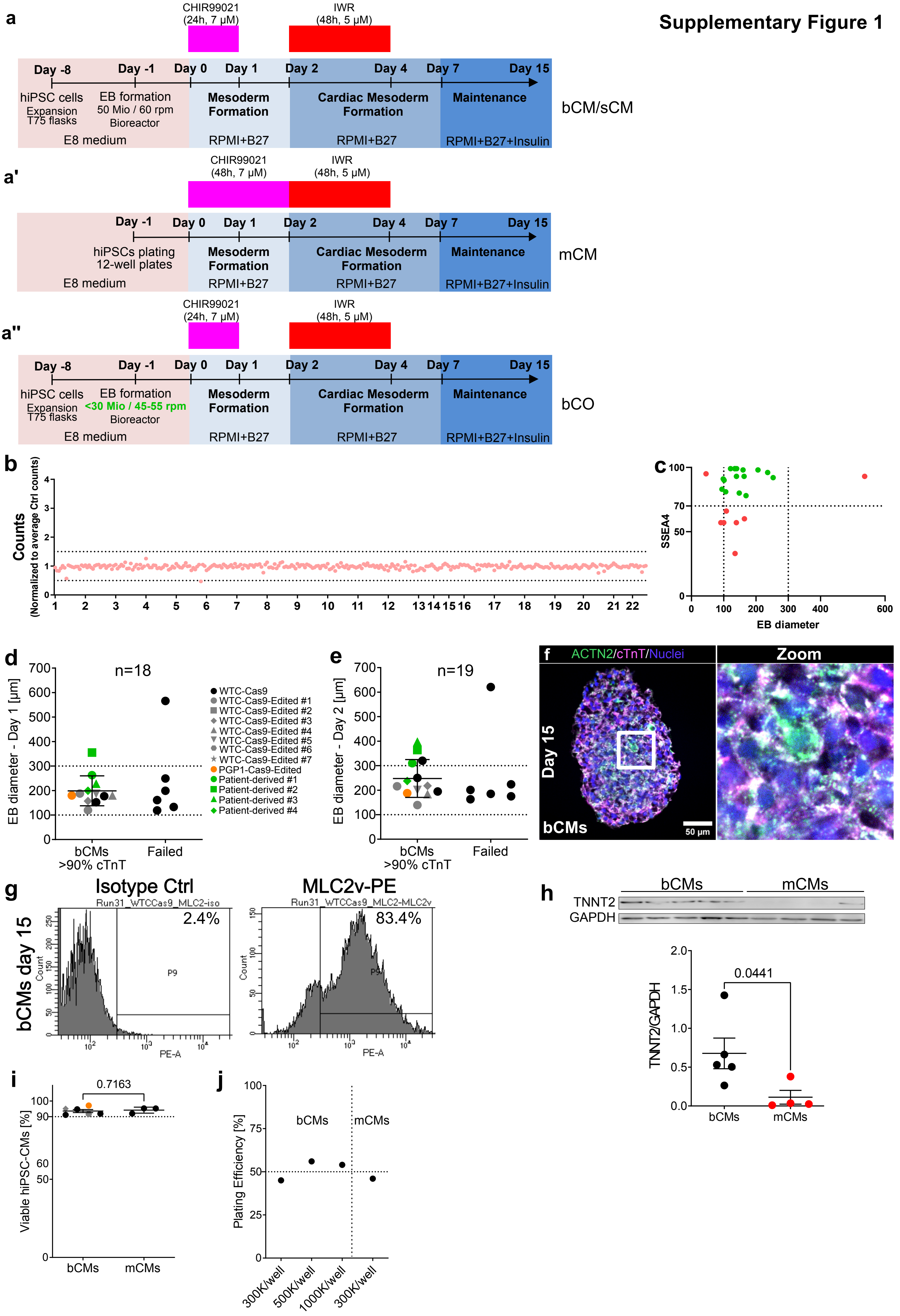

Supplementary Figure 1. Cardiac differentiation protocols. (a) Schematic of cardiac differentiation protocols for suspension culture, monolayer culture, and bioreactor organoid culture. (b) Digital karyotyping results of WTC control hiPSCs at passage 56 (x-axis shows chromosome number). (c) Evaluation of EB diameters and SSEA4 pluripotency (Fig. 1b) showing successfully differentiated bCMs (>90% TNNT2+; green circles) and failed (<90% TNNT2+, red circles) differentiations. (d-e) EB diameter at day 1 (d) or 2 (e) and relationship to bioreactor differentiation outcome. Differentiations with ≤ 90% TNNT2+ cells were classified as "Failed". (f) Representative cryosection of an EB at day 15 stained for TNNT2, sarcomeric alpha actinin (ACTN2) and nuclei (Hoechst 33342). Boxed region is enlarged at right. Bar, 50 µm. (g) Flow cytometry analysis of freshly dissociated bCMs stained for ventricular myosin light chain (MLC2v; n=1 differentiation). (h) Quantification of western blotting experiment stained for TNNT2 and GAPDH using dd15 bCM and mCM samples . Quantification shown below (bCMs n=4 differentiations; mCMs n=3 differentiations). (i) Quantification of viable hiPSC-CMs after cryo-recovery using Trypan blue for bCMs (n=6) and mCMs (n=3) differentiations. (j) Plating efficiency evaluated in 12-well plates at different densities for bCMs (n=1 differentiation; 3 cryovials) and mCMs (n=1 differentiation, 1 cryovial). Welch's unpaired t-test. Data are expressed as mean ± SEM. Source data are provided as a Source Data file.

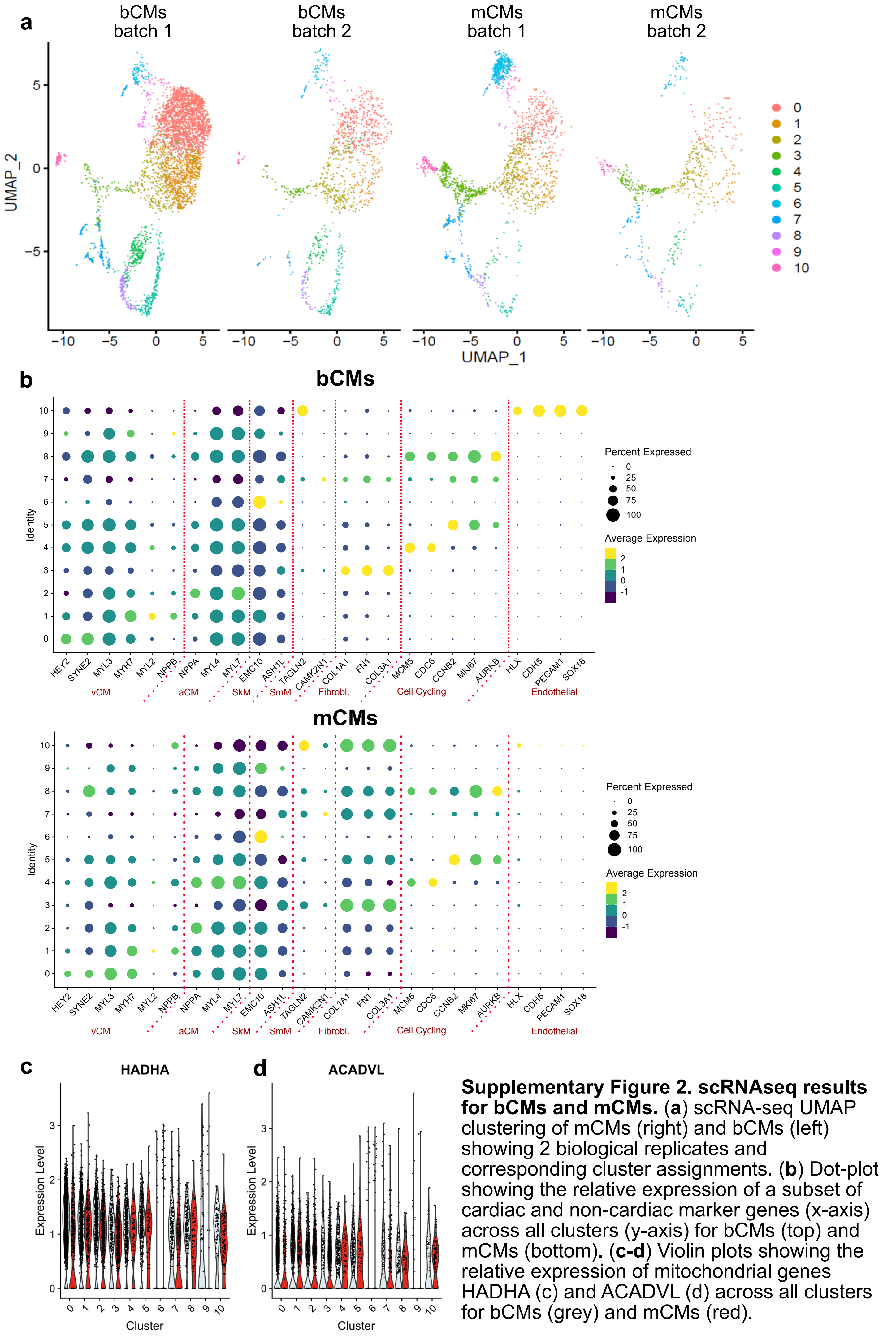

Supplementary Figure 2. scRNAseq results for bCMs and mCMs. (**a**) scRNA-seq UMAP clustering of mCMs (right) and bCMs (left) showing 2 biological replicates and corresponding cluster assignments. (**b**) Dot-plot showing the relative expression of a subset of cardiac and non-cardiac marker genes (x-axis) across all clusters (y-axis) for bCMs (top) and mCMs (bottom). (**c-d**) Violin plots showing the relative expression of mitochondrial genes HADHA (c) and ACADVL (d) across all clusters for bCMs (grey) and mCMs (red).

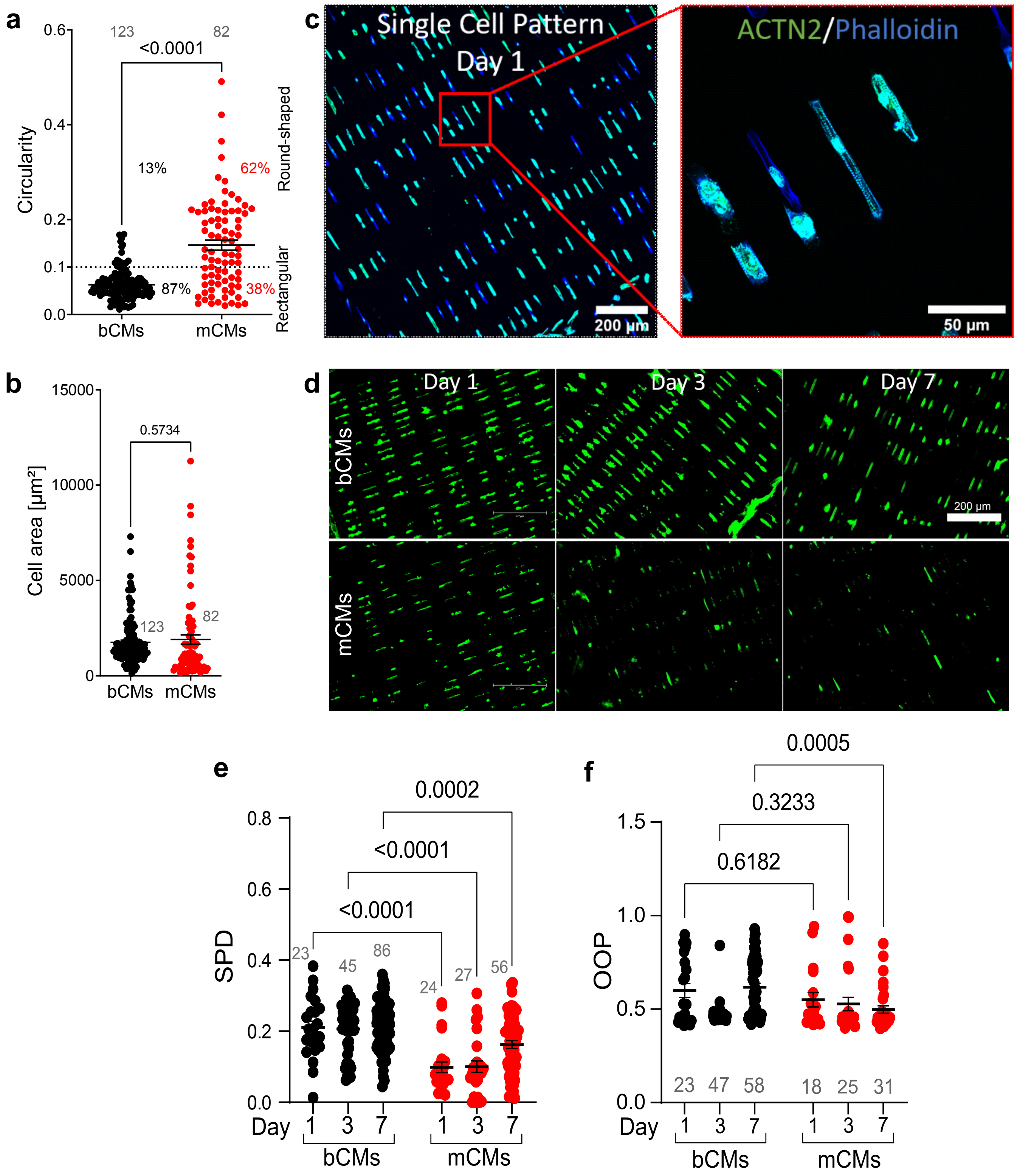

Supplementary Figure 3. Morphological characterization of bCMs and mCMs. Morphology of cryo-recovered bCMs and mCMs was characterized in 2D culture. (a-b). Circularity and area of unpatterned cells after 7 days in culture. Distribution of elongated- (< 0.1 Circularity) and round-shaped (≥ 0.1 Circularity) hiPSC-CMs is indicated in the graph (a). Measurements of 123 bCMs and 82 mCMs obtained from 3 independent differentiation batches. Welch's unpaired t-test. (c-f) Morphological characterization of bCMs and mCMs plated on 7:1 ECM rectangles. (c) Representative image of bCMs fixed 1 day after plating on patterned substrates. Bar, 200 µm (left) or 50 µm (right). (d) Representative images used to quantify coverage of micropatterns by bCMs and mCMs. Bar, 200 µm. (e-f) Sarcomere organization of patterned bCMs or mCMs. ACTN2 and phalloidin-stained cells were analyzed to determine their sarcomere packing density (SPD) and orientational order parameter (OOP; see Methods). Two-way ANOVA with Šidák´s post-test. Data are expressed as mean ± SEM. Grey numbers indicate cells analyzed. Source data are provided as a Source Data file.

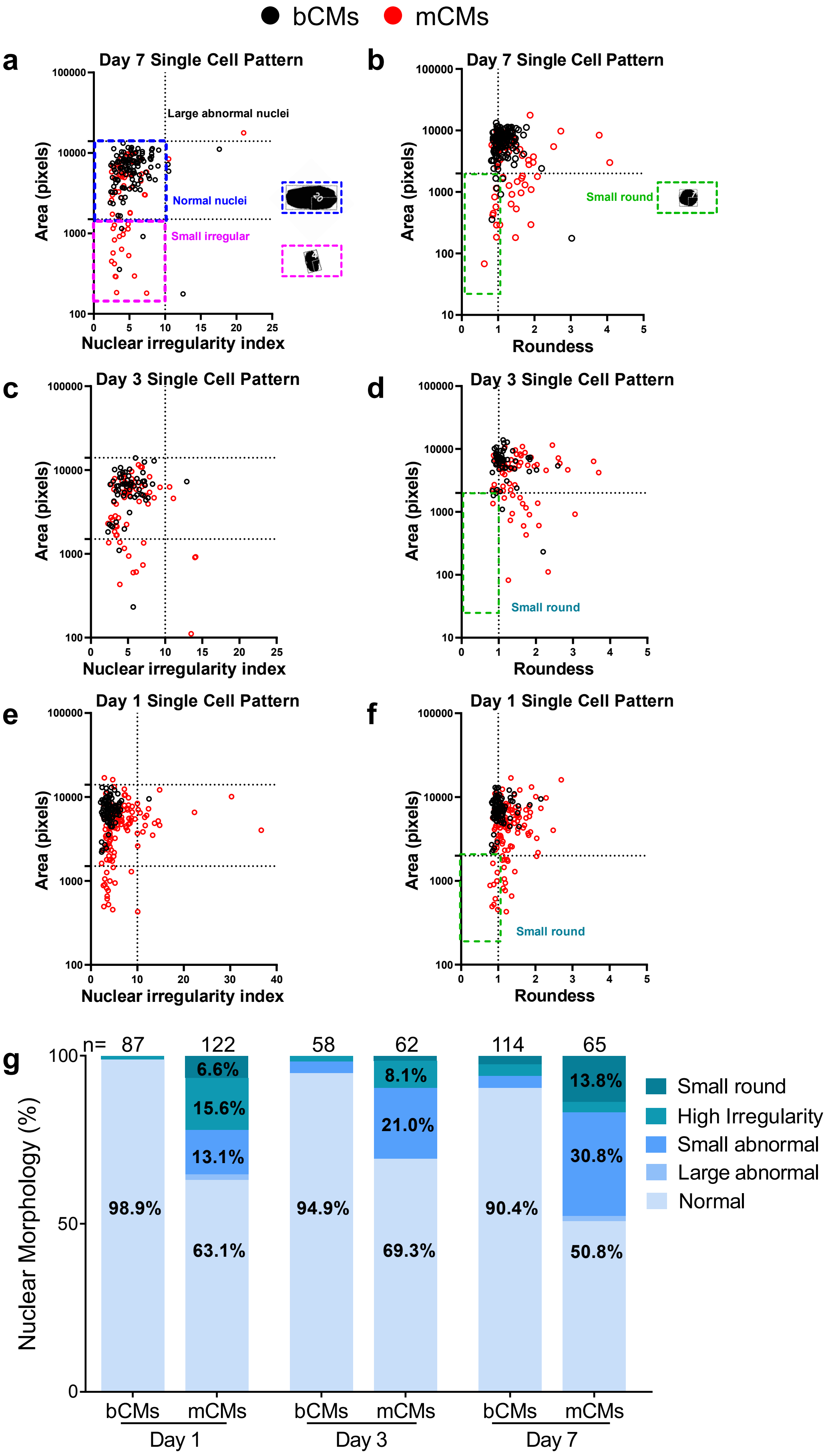

Supplementary Figure 4. Nuclear morphology of micropatterned bCMs and mCMs. bCMs and mCMs were cryo-recovered and plated on single cell ECM rectangles. Unbiased analysis of nuclear morphology was performed using the Nuclear Morphometric Analysis plugin for ImageJ at day 7 (**a-b**), 3 (**c-d**) and 1 (**e-f**). Blue boxes indicated nuclei with normal morphology, violet boxes nuclei with small irregular morphology and green boxes nuclei with small round morphology characteristic of apoptosis. Representative examples are shown to the right of plots a-b. (**g**) Quantification of normal and abnormal nuclei from day 1 to 7. Chi-square test: Day 1, $p<0.0001$; Day 3, $p<0.0001$; Day 7, $p<0.0001$. Number of nuclei analyzed is indicated above bars. Source data are provided as a Source Data file.

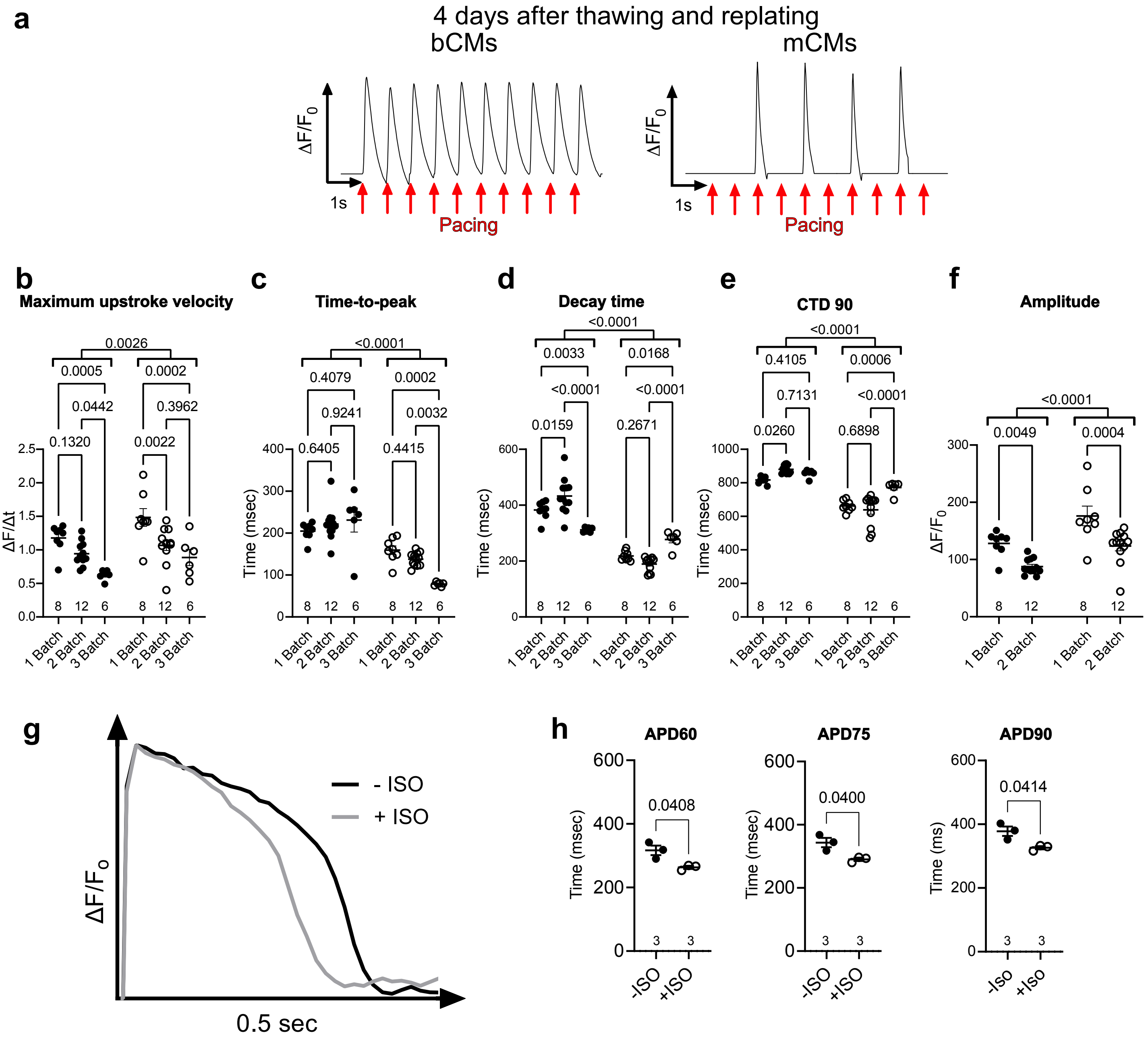

Supplementary Figure 5. Physiological characterization of bCMs. a. Representative Ca^{2+} transients of bCMs (left) and mCMs (right) paced at 1 Hz after 4 days in culture. Pacing signals are indicated by the red arrows. (**b-f**) Cryo-recovered bCM Ca^{2+} transients were recorded with 1 Hz electrical pacing. Cells were loaded with Fluo-4 and optically recorded. Maximum upstroke velocity (b), time-to-peak (c), decay time (d), calcium transient duration (CTD) 90 (e) and amplitude (h) in the presence (empty circles) or absence (filled circles) of 1 µM isoproterenol (ISO) were compared using one-way ANOVA with Šidák´s post-test. Number of wells quantified is indicated along the bottom of the plot. (**g-h**) Cryo-recovered bCM action potentials were recorded with 1 Hz electrical pacing. Cells were loaded with Fluovolt and optically recorded. (g) Representative action potential of bCMs with and without isoproterenol (ISO) treatment. h, Quantification of bCM action potential duration (APD) at 60%, 75%, or 90% recovery, in the presence or absence of 1 µM ISO. Paired t-test. Data are expressed as mean ± SEM. Source data are provided as a Source Data file.

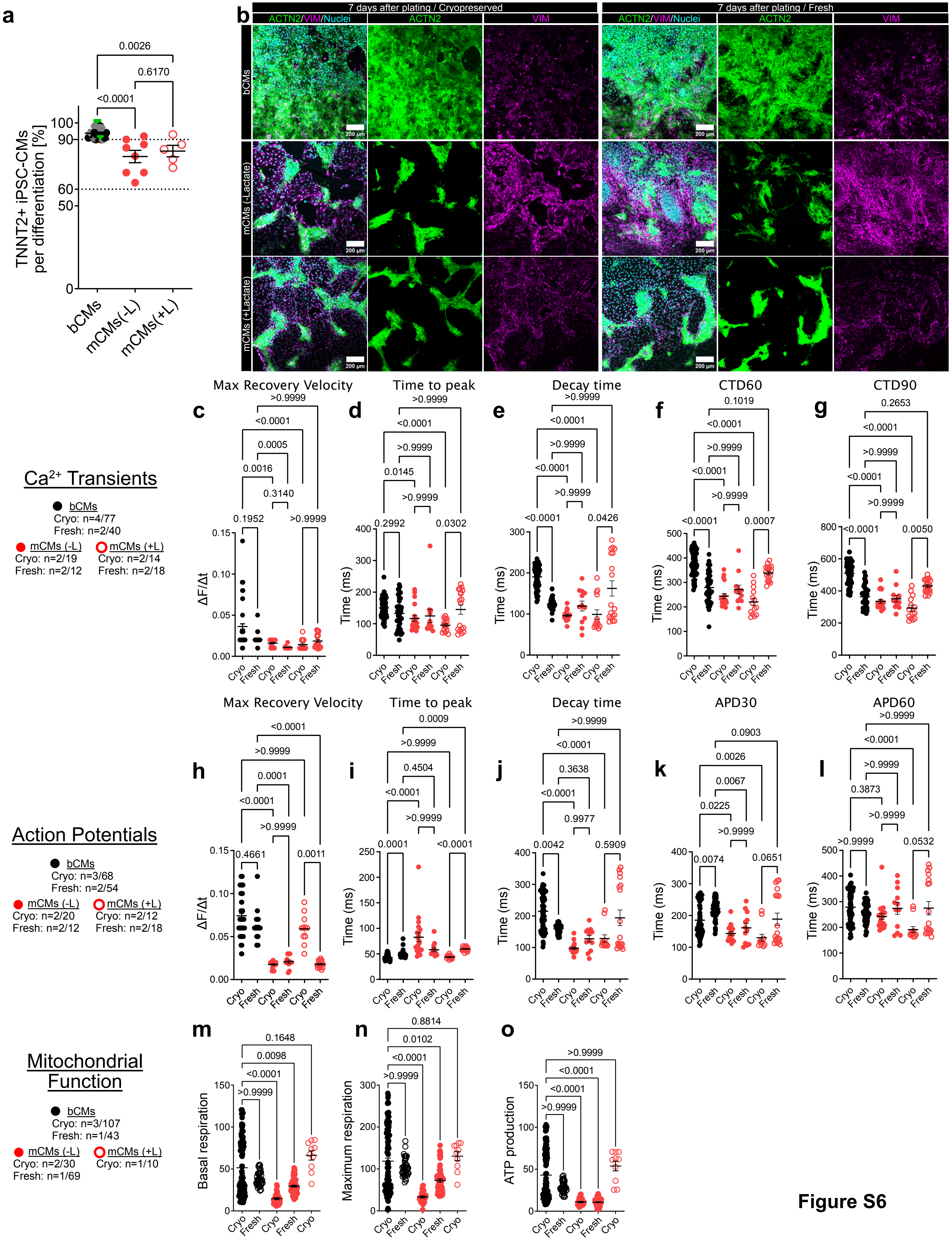
a
TNNT2+ iPSC-CMs per differentiation [%]
0.0026
0.6170
<0.0001
bCMs
mCMs(-L)
mCMs(+L)
b
7 days after plating / Cryopreserved
7 days after plating / Fresh
ACTN2/VIM/Nuclei
ACTN2
VIM
bCMs
mCMs (-Lactate)
mCMs (+Lactate)
200 µm
Ca2+ Transients
bCMs
Cryo: n=4/77
Fresh: n=2/40
mCMs (-L)
Cryo: n=2/19
Fresh: n=2/12
mCMs (+L)
Cryo: n=2/14
Fresh: n=2/18
c
Max Recovery Velocity
ΔF/Δt
d
Time to peak
e
Decay time
f
CTD60
g
CTD90
Time (ms)
Cryo
Fresh
Action Potentials
bCMs
Cryo: n=3/68
Fresh: n=2/54
mCMs (-L)
Cryo: n=2/20
Fresh: n=2/12
mCMs (+L)
Cryo: n=2/12
Fresh: n=2/18
h
Max Recovery Velocity
i
Time to peak
j
Decay time
k
APD30
l
APD60
Mitochondrial Function
bCMs
Cryo: n=3/107
Fresh: n=1/43
mCMs (-L)
Cryo: n=2/30
Fresh: n=1/69
mCMs (+L)
Cryo: n=1/10
m
Basal respiration
n
Maximum respiration
o
ATP production

Figure S6

Supplementary Figure 6. Physiological characterization of fresh and cryo-recovered bCMs and mCMs. mCMs were either treated with lactate (+L) or not (-L). Cells were analyzed fresh or 7 days after cryo-recovery (“cryo”). (a) Percentage of TNNT2+ cells in bCM, mCM-L, and mCM+L cultures. mCMs were on average already at least 80% TNNT2+ prior to lactate treatment. (b) Representative images of cryopreserved and freshly plated hiPSC-CMs stained for actinin 2 (ACTN2), vimentin (VIM) and Hoechst 33342 for nuclei. Images illustrate formation of a confluent cardiomyocyte layer for cryo and fresh bCMs in contrast to mCMs. Bar, 200 µm. (c-g) Ca^{2+} transients of Fluo-4 loaded cells were recorded with 1 Hz pacing. Two-way ANOVA with Šidák´s post-test. (h-l) Action potentials were recorded from Fluovolt-loaded cells paced at 1 Hz. (m-o) Mitochondrial stress test of bCMs, mCMs-L, and mCMs+L. Quantitative comparison of normalized oxygen consumption rates (OCR) corresponding to basal respiration (m), maximal respiratory capacity (n), and ATP production (o). Kruskal-Wallis with Dunn's multiple comparison test.. Number of differentiation batches/wells quantified is indicated in the plots. Data are expressed as mean ± SEM. Data from panel a was replotted from Fig. 1e to facilitate comparisons. Source data are provided as a Source Data file.

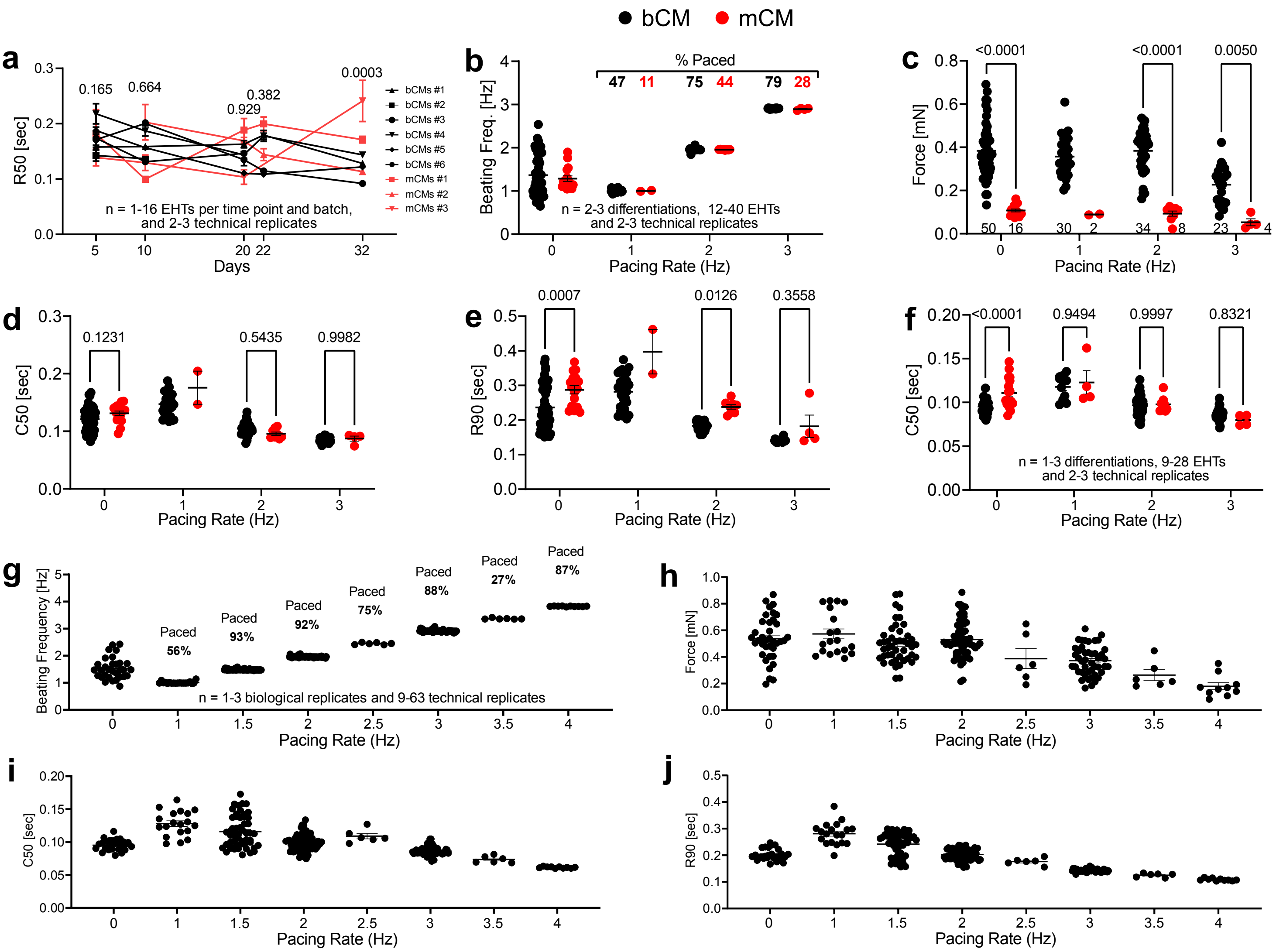

Supplementary Figure 7: Characterization of EHTs assembled from bCMs or mCMs. EHTs assembled from cryo-recovered bCMs or mCMs were recorded in culture medium at 37°C from day 5 to day 32. **a.** Analysis of the 50% relaxation time (R50) of spontaneously beating EHTs. (**b-f**) Analysis of EHTs in culture medium (b-e) or Tyrode solution (f) without pacing (0 Hz) or with 1-3 Hz pacing. b, EHT beat frequency in response to pacing. Only EHTs captured by pacing are shown. The percent of EHTs captured at each pacing rate is indicated. Two-way ANOVA with Sidak´s post-test. (**g-j**) Extended graphs of Fig. 4f-h and Fig. S7f showing frequency (g), force (h), C50 (i) and R90 (j) measurements of bCM EHTs in Tyrode solution at 37°C and paced at 1, 1.5, 2, 2.5, 3, 3.5 and 4 Hz. Data are expressed as mean ± SEM. Source data are provided as a Source Data file.

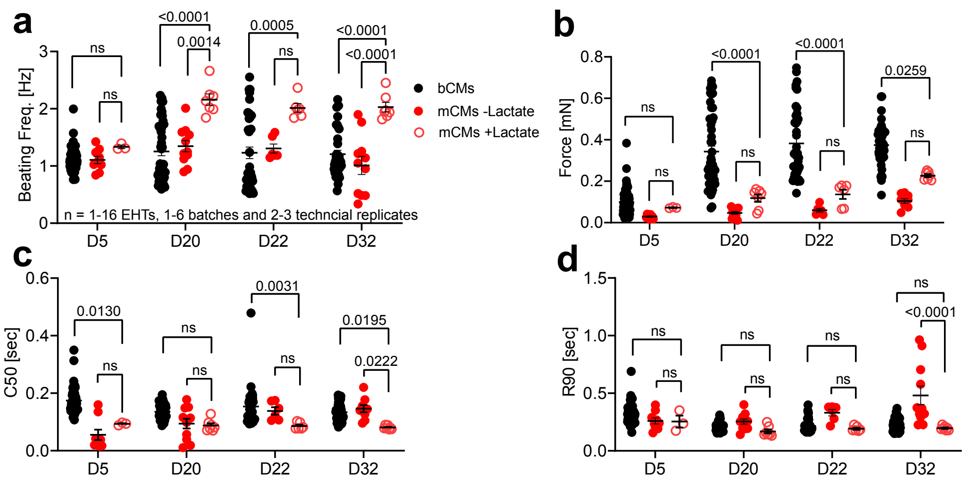

Supplementary Figure 8: Comparison of 3D engineered heart tissues (EHTs) constructed with cryo-recovered bCMs, mCMs-L, and mCMs+L. Spontaneously beating TNNI1-GFP mCM+L EHTs (n=1 differentiation, 3 EHTs, 2-3 technical replicates) were recorded in culture medium at 37°C from day 5 to day 32 and compared to bCMs and mCM EHT data (Fig. 4b-e). Analyses of baseline frequency (a), force (b), time to 50% contraction (C50; c) and 90% relaxation (R90; d) showed greater force generation in bCMs than mCM± EHTs. C50 was shorter in mCMs+L, although these EHTs also had a significantly higher beat rate. R90 was comparable between bCMs and mCMs+L. Two-way ANOVA with Šidák´s post-test of pooled values for each time point. Source data are provided as a Source Data file.

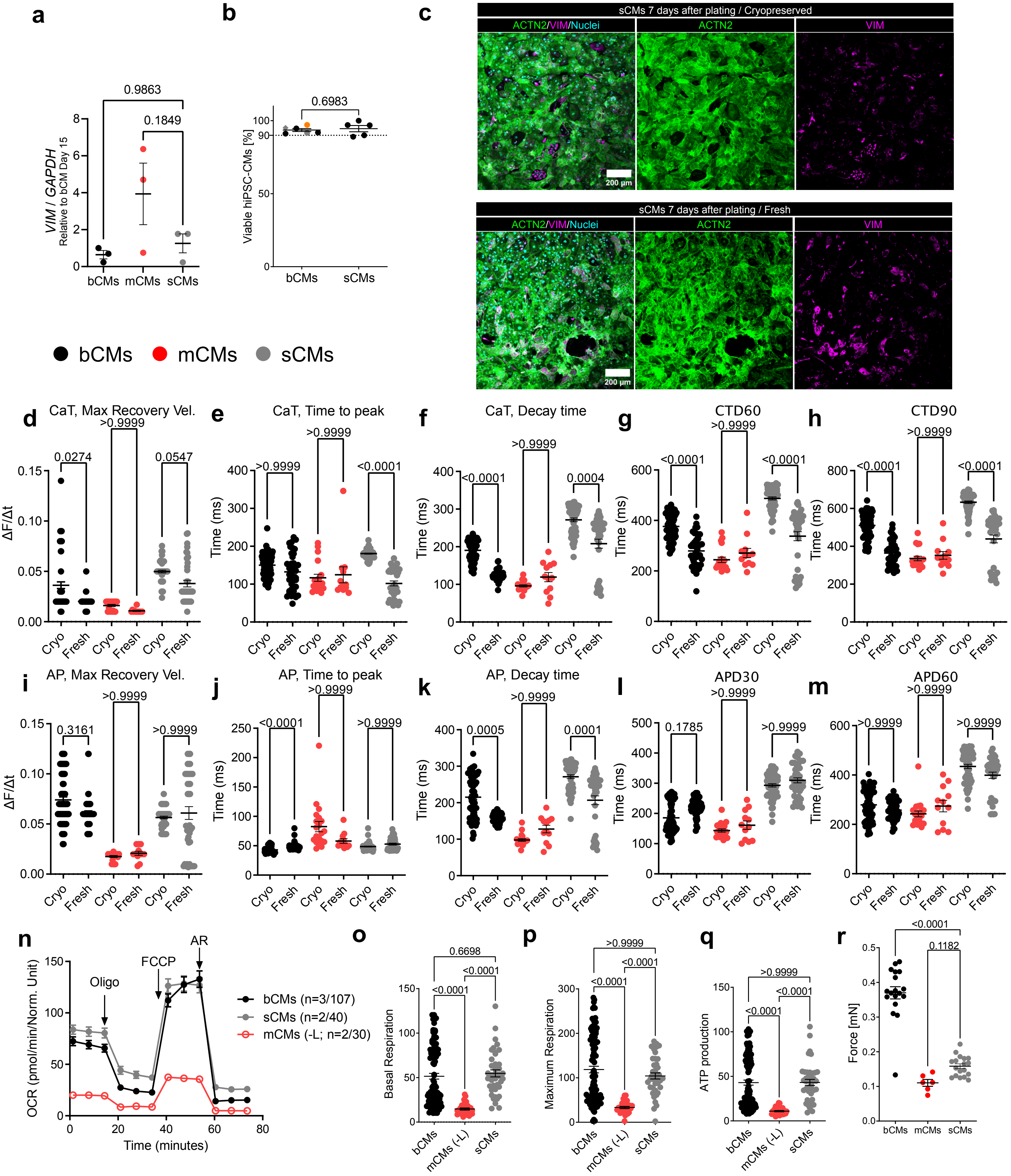
a
b
c
0.9863
0.1849
VIM / GAPDH
Relative to bCM Day 15
bCMs mCMs sCMs
0.6983
Viable hiPSC-CMs [%]
bCMs sCMs
sCMs 7 days after plating / Cryopreserved
ACTN2/VIM/Nuclei
ACTN2
VIM
200 µm
sCMs 7 days after plating / Fresh
bCMs mCMs sCMs
d CaT, Max Recovery Vel.
e CaT, Time to peak
f CaT, Decay time
g CTD60
h CTD90
ΔF/Δt
Time (ms)
Cryo Fresh Cryo Fresh Cryo Fresh
i AP, Max Recovery Vel.
j AP, Time to peak
k AP, Decay time
l APD30
m APD60
n
Oligo
FCCP
AR
OCR (pmol/min/Norm. Unit)
Time (minutes)
bCMs (n=3/107)
sCMs (n=2/40)
mCMs (-L; n=2/30)
o
Basal Respiration
p
Maximum Respiration
q
ATP production
bCMs mCMs (-L) sCMs
r
Force [mN]
bCMs mCMs sCMs

Supplementary Figure 9. Application of the optimized suspension culture differentiation protocol to spinner flasks. (a) RT-qPCR analysis of expression of non-cardiomyocyte marker gene VIM at dd15. n=number of differentiations: bCMs (n=3;); mCMs (n=3); sCMs (n=3). (b) Quantification of viable cells after cryo-recovery. Viability was determined by Trypan blue staining of bCMs and sCMs. Number of differentiations: bCM, 6; sCM, 5. Welch’s unpaired t-test. (c) Representative images illustrate formation of a confluent CM layer for fresh and cryo-recovered sCMs. Bar, 200 µm. (d-h). Ca2+ transients were recorded with 1 Hz pacing using Fluo-4. n=number of differentiations; number of wells for sCMs: cryo: n=2/58; fresh: n=3/40. Action potentials were recorded from Fluovolt-loaded paced at 1 Hz. Maximum upstroke velocity (i-m). Action potentials were recorded with 1 Hz pacing using Fluovolt. n=number of differentiations; number of wells for sCMs: cryo: n=2/59; fresh: n=3/40. Kruskal-Wallis with Dunn's multiple comparison test. Data in d-m are compared to mCM-L and bCMs from Figs. 3 and S6. (n) Mitochondrial function of cryopreserved CMs was assessed by measuring normalized oxygen consumption rates (OCR) and treating cells with Oligomycin (Oligo), FCCP and Antimycin A/Rotenone (AR) (arrows) to yield quantitative estimates of basal respiration (o), maximal respiratory capacity (p), and OCR attributable to ATP production (q). Number of differentiation batches/wells quantified is indicated in the plots. Two-way ANOVA with Šidák´s post-test. (r) Force of spontaneously beating EHTs 32 days after fabrication for bCMs, mCMs-L and sCMs replotted from Figs. 4c and 5w. n=number of differentiations; number of EHTs for sCMs: n=1/10. Data are expressed as mean ± SEM. Source data are provided as a Source Data file.

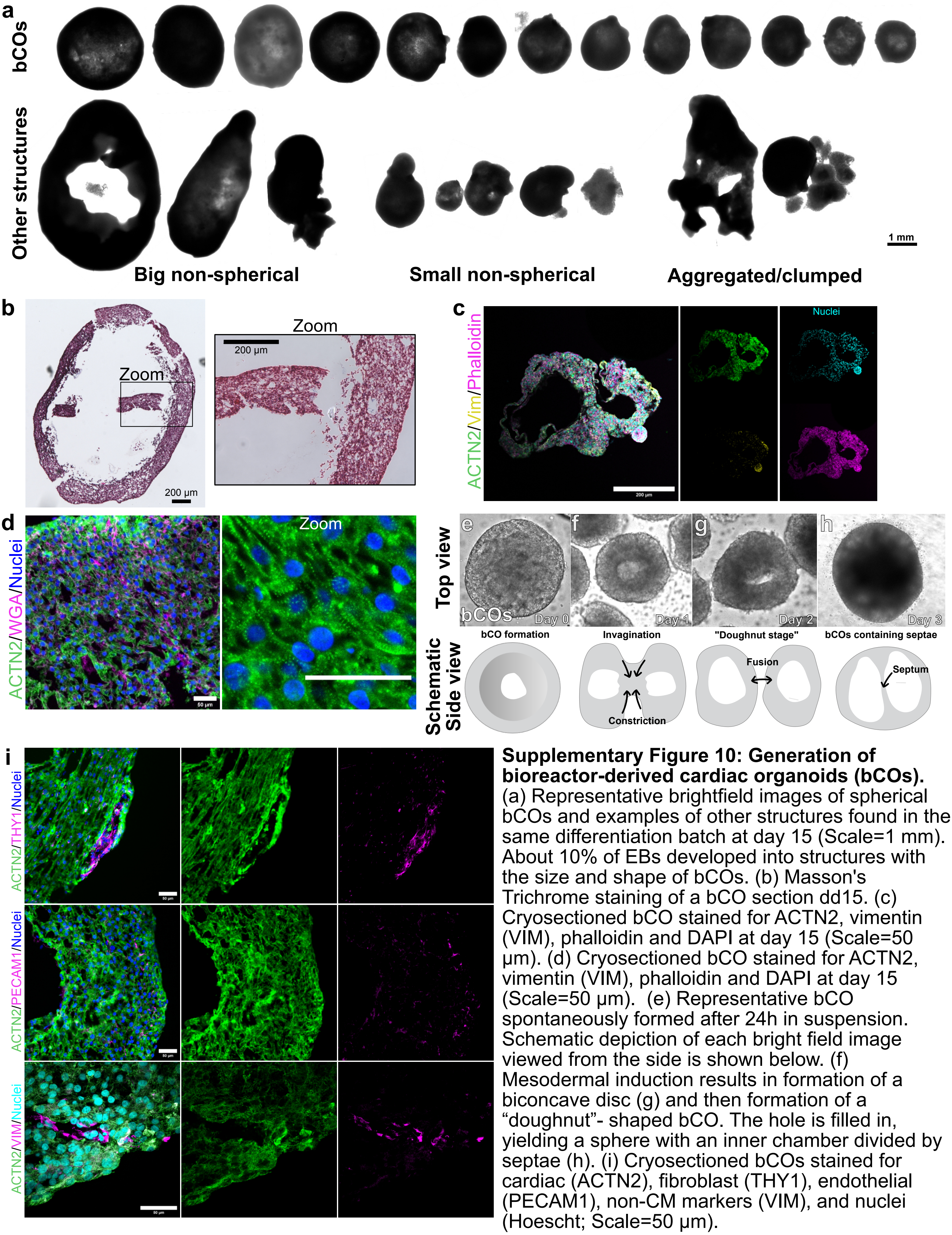

Supplementary Figure 10: Generation of bioreactor-derived cardiac organoids (bCOs). (a) Representative brightfield images of spherical bCOs and examples of other structures found in the same differentiation batch at day 15 (Scale=1 mm). About 10% of EBs developed into structures with the size and shape of bCOs. (b) Masson's Trichrome staining of a bCO section dd15. (c) Cryosectioned bCO stained for ACTN2, vimentin (VIM), phalloidin and DAPI at day 15 (Scale=50 µm). (d) Cryosectioned bCO stained for ACTN2, vimentin (VIM), phalloidin and DAPI at day 15 (Scale=50 µm). (e) Representative bCO spontaneously formed after 24h in suspension. Schematic depiction of each bright field image viewed from the side is shown below. (f) Mesodermal induction results in formation of a biconcave disc (g) and then formation of a “doughnut”- shaped bCO. The hole is filled in, yielding a sphere with an inner chamber divided by septae (h). (i) Cryosectioned bCOs stained for cardiac (ACTN2), fibroblast (THY1), endothelial (PECAM1), non-CM markers (VIM), and nuclei (Hoescht; Scale=50 µm).

Supplementary Table 1. 2D and 3D protocols for iPSC differentiation to iPSC-CMs.

The table summarizes key features of selected iPSC-CM differentiation protocols and the properties of the resulting cells.

Reference	Lian	Burridge	Chen	Kempf (2014, 2015)	Breckwoldt	Correia		Halloin	Hamad		Kahn Krell		Prondzynski (this study)				
Year	2012; 2013	2014	2015	2014, 2015	2017	2018		2019	2019		2021		2023				
Format	ML	ML	Spinner flask	Bioreactor	Spinner and Suspension flask	ML	Aggrewells and Oribtal suspension culture	Spinner flask, Bioreactor, Bioblock	ML	Bioreactor	ML	Shaker flask	ML	ML	Bioreactor	Spinner flask	Spinner flask (3.8x scaling)
Cell lines tested	6	11	3	4	8	5		3	3		1		1	1	14	2	1
Master cell bank	No	No	No	No	No	No		No	No		No		yes				
Pluripotency	OCT4	SSEA4, OCT4, TRA-1-60	Oct4, SSEA4, TRA-1-60	SSEA3, TRA-1-60	SSEA3, TRA-1-60	No		No	No		SSEA4, TRA-1-60, SOX2		SSEA4				
Rounds per minute (RPM)	-	-	35, 45, 55	60	40	-	90	60	-	60	-	55	-	-	60	60	60
Culture Volume (mL)	-	-	125, 500, 1000	100	46	-	-	150, 350-500	-	125	-	2.5, 30	-	-	100	100	380
EB size D0 (µM)	-	-	~160–280	~531	-	-	100-300	~128	-	-	-	~242	-	-	~158	~88	-
Wnt activation	CHIR (12 µM)	CHIR	CHIR (6, 12, 18, 24 µM)	CHIR (7.5 µM)	BMP4, Activin A, bFGF	CHIR (12 µM), Activin A, Ascorbic Acid	CHIR (12 µM), Activin A, Ascorbic Acid	CHIR (5, 7.5 µM)	CHIR (8 µM)		CHIR (6 µM)		CHIR (7 µM)				
Wnt inhibition	IWP2, IWP4, shRNA	Wnt-C59	IWP4	IWP	DS-I-7, IWR	IWR-1	IWR-1	IWP2	IWP2, XAV939		IWR		IWR				
Wnt inhibition day	3	2	2 and 3	3	3	1	1	1 and 3	3		3		2				
1st beating day	8	-	8	6-7	8	6-8		-	7		-		7		5		
CM enrichment	No	Metabolic enrichment	No	No	No	No		No	No		Metabolic enrichment		Metabolic enrichment	No			
Yield (M cells/ml)	~4 (H)	~3 (H)	~1.5-2.0	~0.5	-	-		~1	~1.8	~0.72	~1	~1.5	-	~0.28	~1.24	~1.79	~3.4
Yield (output CM/input iPSC)	-	-	4.8-5.3	-	0.9	-		2	2	2.2	-	?	-	~1.68	~2.5	~3.58	~6.9
TNNT2+ (%)	>80	>80-95	>20-80	>80	>70	>70	>80	>90	>90	>80	>90		>70	>70	>90	>88	>90
ventricular CMs (%) (variable assessment times)	-	25 (B)	47 (A)	85 (A)	-	-		95 (A)	65 (B)		-		-	44 (C)	67 (C)	-	-
Cryopreservation medium	-	-	CryoStor CS10	-	FBS + DMSO	-		-	-	-	-	-	Stem cell cardiomyocyte freezing medium				
Cryopreservation method	-	-	Controlled rate freezer	-	Isopropanol filled cryobox	-		-	-	-	-	-	Controlled rate freezer				
Viability after cryopreservation (%	No	No	~ 85.8	No	~ 82	No		No	No		No		No	>90	>90	>90	>90
Plating efficiency (%)	No	No	No	No	No	No	No	No	No	No	No	No	No	~ 46	~ 51	No	No
Gene expression	No	Yes	Yes	Yes	No	Yes	Yes	Yes	Yes		Yes		No	Yes			-
Unpatterned CM morphology	Yes	Yes	Yes	Yes	No	-		Yes	No		Yes		No	Yes		No	-
Cell Area (µm2)	No	No	No	No	No	-		~1400	No		No		No	~1905	~1752	No	-
Patterned CM morphology	No	No	No	No	No	-		No	No		No		No	Yes		No	-
AP dVmax/dt (V/s)	No	~8 (D)	~45 (D)	No	No	-		No	No		No		No				-
APD50 (ms)	No	~140 (D)	~250 (D)	~600 (D)	No	~300 (G)	~350 (G)	~2000 (D)	~140 (G)	No	No		No				-
APD60 (ms)	No	?	No	No	No	-		No	No		No		~191 (I); ~275 (J)	~242 (I); ~274 (J)	~279 (I); ~260 (J)	~434 (I); ~399 (J)	-
APD90 (ms)	No	~180 (D)	~350 (D)	No	No	~380 (G)	~430 (G)	No	No		No		~274 (I); ~340 (J)	~365 (I); ~384 (J)	~346 (I); ~318 (J)	~506 (I); ~462 (J)	-
Ca Time-to-Peak (ms)	No	No	No	No	No	-		No	~400	No	No		~95 (I); ~145 (J)	~116 (I); ~124 (J)	~150 (I); ~133 (J)	~180 (I); ~104 (J)	-
CaTD60 (ms)	No	No	No	No	No	-		No	No		No		~220 (I); ~338 (J)	~244 (I); ~270 (J)	~376 (I); ~283 (J)	~487 (I); ~339 (J)	-
CaTD90 (ms)	No	No	No	No	No	-		No	No		No		~292 (I); ~430 (J)	~335 (I); ~352 (J)	~510 (I); ~364 (J)	~633 (I); ~443 (J)	-
Ca Amplitude (ms)	No	No	No	No	No	-		-	No		No		~1.8 (I); ~3.1 (J)	~2.2 (I); ~1.3 (J)	~7.8 (I); ~2.1 (J)	~11.3 (I); ~6.4 (J)	
Metabolic Assesment	No	No	No	Monitoring of dissolved oxygen and pH	No	Glucose consumption, lactate production, amino acid production,		Monitoring of dissolved oxygen and pH	No		No		Seahorse				-
EHT -- force (mN)	No	No	No	~0.4 (E)	~0.15 (F)	-		~1.3 (E)	No		No		~0.226 (I,K)	~0.10 (I,K);~0.17 (I,F)	~0.37 (I,K);~0.53 (I,F)	~0.158 (I,K	-
EHT -- C50 (sec)	No	No	No	No	No	-		No	No		No		~0.08 (I,K)	~0.14 (I,K);~0.13 (I,F)	~0.13 (I,K);~ 0.12 (I,F)	~0.12 (I,K	-
EHT -- R90	No	No	No	No	No	-		No	No		No		~0.19 (I,K)	~0.28 (I,K);~0.23 (I,F)	~0.23 (I,K);~ 0.20 (I,F)	~0.23 (I,K)	-

-' Information not obtained/reported. (A) Whole cell patch clamp. (B) FACS. (C) Single cell RNA sequencing (Normalized to CM population). (D) Ventricular sublineages paced at day 18-27. (E) Bioartificial cardiac tissue at day 21 (Halloin, supplemented with human fibroblasts). (F) Tyrode Solution. (G) Day 33. (H) Calculated from surface area (0.18 ml/cm^2). (I) Cryopreserved hiPSC-CMs at day 22. (J) Fresh hiPSC-CMs at day 22. (K) RPMI+B27 plus Insulin. (R) Day 35

Supplementary Table 2. Estimate of cost of materials for monolayer or bioreactor iPSC-CM differentiation. Labor costs are excluded. Monolayer costs are for 125 million cells. 100 ml bioreactor culture, or 125M monolayer cells. Yield is about 0.28 M/ml mCM vs 1.23 M/ml bCM. Yield per input iPSC is about 1.7 (mCM) vs 2.5 (bCM). Hardware cost of a bioreactor with 4 independent chambers is approx. $150,000. 4 position spinner flask system costs approx. $5,000.

Item Name	Amount	Item Number	List Price ($)	Bioreactor run	Cost per Bioreactor run ($)	Monolayer run for 125 Mio hiPSC-CMs)	Cost per Monolayer run ($)
**Tissue Culture Flask T80 50 Unit**	50x	178905	205	3x flasks	12.3	2x flasks	8.2
**Pluronic F-127**	1 kg (1% (w/vol))	24040032	274.04	80 mL	0.22	20 mL	0.05
**Geltrex LDEV-Free, hESC-Qualified (5 mL)**	500 mL (1:100 dilution)	A1413302	293	30 mL	17.58	170 mL	99.65
**12-well plates**	50x	877229	194.5	-	-	15x plates	58.35
**Essential 8 Medium**	500 mL	A1517001	234	170 mL	80	210 mL	98.31
**Versene Solution**	100 mL	15040-066	12.27	15 mL	1.8	10 mL	1.22
**RPMI 1640 Medium, GlutaMAX Supplement**	10x 500 mL	61870-127	289	800 mL	46.24	1600 mL	92.48
**B27 SUPPLEMENT (50X)**	10 mL	A1895601	146	16 mL	237.25	24 mL	474.5
**ROCK INHIBITOR Y27632**	10 mg (3.12 mL at 10mM)	NC0791122	205	130 µL	8.54	170 µL	11.17
**IWR-1 ≥98% (HPLC)**	5 mg (2.44 mL at 5mM)	I0161	81.9	100 µL	3.35	225 µL	7.55
**CHIR99021**	10 mg (2.15 mL at 10mM)	72052	269	70 µL	8.76	148.5 µL	18.6
**Collagenase II**	1 g (625 mL at 200units/mL)	NC9693955	195	60 mL	18.73	150 mL	47.56
**N-BENZYL-P-TOLUENESULFONAMIDE**	25 g (3200 mL at 10mM)	TCB3082-25G	418.4	60 uL	0.007	150 uL	0.019
**Deoxyribonuclease II Type V**	196 mg (100 mL at 1500 units/mL)	D8764-150KU	465	360 uL	1.67	900 uL	4.18
**STEMdiff™ Cardiomyocyte Freezing Medium**	50 mL	5030	160	25 mL	80	25 mL	80
**PBS 1X, PH 7.4 10 X 500ML**	500 mL	10010049	76.05	100 mL	15.21	100 mL	15.21
**HBSS, calcium, magnesium, no phenol red**	500 mL	14175095	25.9	100 mL	5.18	300 mL	15.54
**Insulin solution human Chemically defined, recombinant, expressed in Saccharomyces cerevisiae, sterile-filtered, BioXtra, suitable for cell culture**	5mL	I9278-5ML	206	~ 500 uL	20.6	~ 750 uL	30.9
**Cryotubes**	450x	12565167N	670.5	25x	11.4	25x	11.4
				Sum	**568.84**	Sum	**1074.89**

Supplementary Table 3. Antibodies used in this study.

Target Protein	Manufacturer	Catalog Number	Lot Number	Dilution	Application
Control-FITC	Miltenyi Biotec	130-113-449	5210504501	1 to 50	Flow cytometry
SSEA4-FITC	Miltenyi Biotec	130-122-918	1322050485	1 to 50	Flow cytometry
TNNT2-FITC	Miltenyi Biotec	130-119-575	5230803551	1 to 50	Flow cytometry
Control-PE	Miltenyi Biotec	130-113-762	5220607743	1 to 50	Flow cytometry
MLC2v-PE	Miltenyi Biotec	130-119-680	1324020702	1 to 50	Flow cytometry
ACTN2	Abcam	AB9465	1058926-5	1 to 200	Immunoflourescence
Phalloidin	Invitrogen	A22287	2326923	1 to 400	Immunoflourescence
Wheat Germ Agglutinin	Invitrogen	W32466	2126807	1 to 400	Immunoflourescence
cTnT	Abcam	ab45932	GR3427142-1	1 to 200	Immunoflourescence
Hoeschet	Life Technologies	H1399	1924446	1 to 500	Immunoflourescence
H2AFX	Cell signaling Technologies	9718S	17	1 to 200	Immunoflourescence
Vimentin	R&D Systems	MAB2105	UUQ0222101	1 to 200	Immunoflourescence
THY1	Abcam	ab133350	GR3368588-12	1 to 200	Immunoflourescence
CD31 (PECAM1)	Invitrogen	17-0319-42	2507946	1 to 200	Immunoflourescence
Alexa 488 RB	Invitrogen	A32790	VC296619	1 to 400	Immunoflourescence
Alexa 488 MS	Invitrogen	A21131	1964395	1 to 400	Immunoflourescence
Alexa 555 RB	Invitrogen	A31572	2339822	1 to 400	Immunoflourescence
Alexa 555 Rat	Invitrogen	A21434	2329406	1 to 800	Immunoflourescence
Alexa 555 MS	Invitrogen	A21045	706307	1 to 400	Immunoflourescence
TNNT2	Life Technologies	MA512960	-	1 to 800	Western Blot
GAPDH	Cell signaling Technologies	2118S	-	1 to 800	Western Blot

Supplementary Table 4. Primers used in this study.

Name	Gene	Direction	5´-3´
Alpha-Actinin 2	*ACTN2*	Forward	GCCATGGAAATCGCTGAGAA
Alpha-Actinin 2	*ACTN2*	Reverse	ATCCTGTTAGCCGCTGTCTC
Glyceraldehyde 3-phosphate dehydrogenase	*GAPDH*	Forward	ATGTTCGTCATGGGTGTGAA
Glyceraldehyde 3-phosphate dehydrogenase	*GAPDH*	Reverse	TGAGTCCTTCCACGATACCA
collagen, type I, alpha 1	*COL1A1*	Forward	AAGAGGAAGGCCAAGTCGAG
collagen, type I, alpha 1	*COL1A1*	Reverse	AGATCACGTCATCGCACAAC
collagen type III alpha 1 chain	*COL3A1*	Forward	AGGGGAGCTGGCTACTTCTC
collagen type III alpha 1 chain	*COL3A1*	Reverse	CGGATCCTGAGTCACAGACA
Vimentin	*VIM*	Forward	GGAGCTACGTGACTACGTCCA
Vimentin	*VIM*	Reverse	GAGAAGTCCACCGAGTCCTG
Myosin heavy chain 7	*MYH7*	Forward	GCTCTGTGTCTTTCCCTGCTGCTC
Myosin heavy chain 7	*MYH7*	Reverse	GCTCCTTCTCTGACTTGCGCAGG
Myosin light chain 2	*MYL2*	Forward	TCATGGACCAGAACAGGGAT
Myosin light chain 2	*MYL2*	Reverse	CTCCCCAAACATTGTGAGGA
Myosin light chain 3	*MYL3*	Forward	CCTTCATGCTGTTCGACCG
Myosin light chain 3	*MYL3*	Reverse	AGGCAGGAAAGTTTCAAAGTCC
Myosin light chain 4	*MYL4*	Forward	AACCTGCCTTTGACCCCAA
Myosin light chain 4	*MYL4*	Reverse	CTCGGCATTGGTAGGGTTCT
Myosin light chain 7	*MYL7*	Forward	GAAGGTGAGTGTCCCAGAGG
Myosin light chain 7	*MYL7*	Reverse	CCTTGTTCACCACCCCTTTG
Hyperpolarization activated cyclic nucleotide gated potassium channel 4	*HCN4*	Forward	GGCAAGTCCAGCACGAAC
Hyperpolarization activated cyclic nucleotide gated potassium channel 4	*HCN4*	Reverse	GCGGAGTCATGCAGGTGT
Platelet And Endothelial Cell Adhesion Molecule 1	*PECAM1*	Forward	GAGTCCTGCTGACCCTTCTG
Platelet And Endothelial Cell Adhesion Molecule 1	*PECAM1*	Reverse	ATCTGGTGCTGAGGCTTGAC
CD90	*THY1*	Forward	AGCATCGCTCTCCTGCTAAC
CD90	*THY1*	Reverse	GCACGTGCTTCTTTGTCTCA

Supplementary Table 5. Software settings for the Grant CRF-1 controlled rate freezer.

ID	Segment	Target Temp	Duration	Ramp Rate	FF Offset	Hold Back	Ready To	Nucleate	Cycle On	Heater	Audible
1	Step	4.00	-	-	-	-	-	-	-	-	off
2	Dwell	-	0:02	-	0.00	-	off	off	on	on	off
3	Rate	-10.00	-	2.00	3.00	off	off	off	on	off	off
4	Dwell	-	0:02	-	3.00	-	off	off	on	off	off
5	Dwell	-	0:05	-	4.50	-	off	off	on	off	on
6	Dwell	-	0:05	-	4.50	-	off	off	on	off	off
7	Rate	-40.00	-	1.00	3.00	off	off	off	on	off	off
8	Rate	-80.00	-	5.00	3.00	off	off	off	on	off	off
9	Dwell	-	0:05	-	4.50	-	off	off	on	off	off
10	End	-	-	-	-	-	-	-	-	-	on
