# Supplementary material for: Efficient and reproducible generation of human iPSC-derived cardiomyocytes and cardiac organoids in stirred suspension systems
Source: Nat Commun. 2024 Jul 15;15:5929. doi: 10.1038/s41467-024-50224-0 (PMC11251028; doi:10.1038/s41467-024-50224-0)
Supplement: Supplementary file 3 — Description of Additional Supplementary Files [file 41467_2024_50224_MOESM3_ESM.pdf]

Description of Additional Supplementary Files

Supplementary Movie Legends

Supplementary Movie 1. Immunofluorescent analysis of a bioreactor-differentiated embryoid body at dd15. Staining was performed against TNNT2 (Magenta), ACTN2 (green) and nuclei (blue).

Supplementary Movie 2. Bioreactordifferentiated hiPSC-CMs at dd15. Scale bar, 250 µm.

Supplementary Movie 3. Monolayerdifferentiated hiPSC-CMs at dd15. Scale bar, 250 µm.

Supplementary Movie 4. Cardiac monolayer differentiation in a 12-well plate at dd10. Scale bar, 250 µm.

Supplementary Movie 5. Bioreactordifferentiated embryoid body beating at dd5. Scale bar, 250 µm.

Supplementary Movie 6. Engineered heart tissue cast with cryopreserved bCMs after 29 days in culture. Scale bar, 1 mm.

Supplementary Movie 7. Engineered heart tissue cast with cryopreserved mCMs after 29 days in culture. Scale bar, 1 mm.

Supplementary Movie 8. Engineered heart tissue cast with cryopreserved bCMs transduced with adenovirus ChR2-YFP after 7 days in culture.

Supplementary Movie 9. Engineered heart tissue cast with cryopreserved bCMs optogenetically paced at 4 Hz after 41 days in culture. Scale bar, 1 mm.

Supplementary Movie 10. Bioreactor-derived cardiac organoid at dd15. Scale bar, 250 µm.

Supplementary Movie 11. Cross-section of a bioreactor-derived cardiac organoid at dd15. Scale bar, 250 µm.

Supplementary Data Legends

Supplementary Data 1. Markers of cell clusters in bCM and mCM hiPSC-CM scRNAseq data. Marker genes of each cell cluster are shown.

Supplementary Data 2. Markers of cell clusters in bCO scRNAseq data.
