# Supplementary material for: Efficient and reproducible generation of human iPSC-derived cardiomyocytes and cardiac organoids in stirred suspension systems
Source: Nat Commun. 2024 Jul 15;15:5929. doi: 10.1038/s41467-024-50224-0 (PMC11251028; doi:10.1038/s41467-024-50224-0)
Supplement: Supplementary file 17 — Reporting Summary [file 41467_2024_50224_MOESM17_ESM.pdf]

nature portfolio

Corresponding author(s): William T. Pu

Last updated by author(s): Mar 27, 2024

Reporting Summary

Nature Portfolio wishes to improve the reproducibility of the work that we publish. This form provides structure for consistency and transparency in reporting. For further information on Nature Portfolio policies, see our Editorial Policies and the Editorial Policy Checklist.

Statistics

For all statistical analyses, confirm that the following items are present in the figure legend, table legend, main text, or Methods section.

n/a	Confirmed	
☐	☒	The exact sample size (*n*) for each experimental group/condition, given as a discrete number and unit of measurement
☐	☒	A statement on whether measurements were taken from distinct samples or whether the same sample was measured repeatedly
☐	☒	The statistical test(s) used AND whether they are one- or two-sided<br>*Only common tests should be described solely by name; describe more complex techniques in the Methods section.*
☐	☒	A description of all covariates tested
☐	☒	A description of any assumptions or corrections, such as tests of normality and adjustment for multiple comparisons
☐	☒	A full description of the statistical parameters including central tendency (e.g. means) or other basic estimates (e.g. regression coefficient) AND variation (e.g. standard deviation) or associated estimates of uncertainty (e.g. confidence intervals)
☒	☐	For null hypothesis testing, the test statistic (e.g. *F*, *t*, *r*) with confidence intervals, effect sizes, degrees of freedom and *P* value noted<br>*Give P values as exact values whenever suitable.*
☒	☐	For Bayesian analysis, information on the choice of priors and Markov chain Monte Carlo settings
☒	☐	For hierarchical and complex designs, identification of the appropriate level for tests and full reporting of outcomes
☒	☐	Estimates of effect sizes (e.g. Cohen's *d*, Pearson's *r*), indicating how they were calculated

Our web collection on statistics for biologists contains articles on many of the points above.

Software and code

Policy information about availability of computer code

Data collection

EHT post movement was recorded using Micro-Manager (https://micro-manager.org/).

Data analysis

Downstream differential expression and clustering analysis for scRNAseq data was performed using the Seurat V.4.0 package, as described in the tutorials (http://satijalab.org/seruat/). Doublets were detected and removed using DoubletFinder (v2.0.3). The resulting subset Seurat objects were normalized using the scTransform workflow and further scaled and normalized the RNA assay in order to perform downstream differential expression analysis and marker visualization utilizing the FindMarkers and FeaturePlot functions on the RNA assay. Uniform manifold approximation and projection (UMAP) was performed and iteratively modified after performing marker gene expression and examining expression of key markers.

Image processing/analysis and quantification of cell size and circularity was performed using Fiji (https://fiji.sc). To assess sarcomere alignment in micropatterned hiPSC-CMs we used previously reported methods from the Disease Biophysics Group (Pasqualini et al. 2015; Stem Cell reports). Morphology of nuclei was assessed using a previously published Fiji plug in (https://www.ufrgs.br/labsinal/nma/) .

Raw data of calcium and voltage imaging of unpatterned hiPSC-CMs was analyzed with CyteSeer software (Vala Biosciences) using custom scripts for analysis of calcium transient kinetics and action potential duration.

EHT post movement was tracked post-hoc using the multi-template matching FIJI plugin (Thomas et al., 2020 BMC Bioiformatics). Twitch force measurements were subsequently measured by applying post deflection to the beam bending theory for a known Young's modulus of the posts, as described in detail elsewhere (Vandenburgh et al., 2008 Muscle Nerve).

For manuscripts utilizing custom algorithms or software that are central to the research but not yet described in published literature, software must be made available to editors and reviewers. We strongly encourage code deposition in a community repository (e.g. GitHub). See the Nature Portfolio guidelines for submitting code & software for further information.

Data

Policy information about availability of data

All manuscripts must include a data availability statement. This statement should provide the following information, where applicable:

- Accession codes, unique identifiers, or web links for publicly available datasets
- A description of any restrictions on data availability
- For clinical datasets or third party data, please ensure that the statement adheres to our policy

Datasets, analysis and study materials will be made available on request to other researchers for purpose of reproducing the results or replicating the procedures. Source data is provided with this paper. scRNAseq data are available at Gene Expression Omnibus (GEO, accession #GSE263372 [https://www.ncbi.nlm.nih.gov/geo/query/acc.cgi?acc=GSE223452]). Source data are provided with this paper.

Human research participants

Policy information about studies involving human research participants and Sex and Gender in Research.

Reporting on sex and gender	NA
Population characteristics	NA
Recruitment	NA
Ethics oversight	NA

Note that full information on the approval of the study protocol must also be provided in the manuscript.

Field-specific reporting

Please select the one below that is the best fit for your research. If you are not sure, read the appropriate sections before making your selection.

☒ Life sciences ☐ Behavioural & social sciences ☐ Ecological, evolutionary & environmental sciences

For a reference copy of the document with all sections, see nature.com/documents/nr-reporting-summary-flat.pdf

Life sciences study design

All studies must disclose on these points even when the disclosure is negative.

Sample size	No sample size calculation was performed.
Data exclusions	No data were excluded.
Replication	Definitions of biological replicates and technical replicates, for iPSC and biochemical assays, are described in detail in the manuscript. The individual results from each biological replicate are displayed in the main figure panels and demonstrate that all experimental findings were able to be reliably reproduced.
Randomization	There was no randomization in this study.
Blinding	Investigators were not blinded.

Reporting for specific materials, systems and methods

We require information from authors about some types of materials, experimental systems and methods used in many studies. Here, indicate whether each material, system or method listed is relevant to your study. If you are not sure if a list item applies to your research, read the appropriate section before selecting a response.

Materials & experimental systems

n/a	Involved in the study	
☐	☒	Antibodies
☐	☒	Eukaryotic cell lines
☒	☐	Palaeontology and archaeology
☒	☐	Animals and other organisms
☒	☐	Clinical data
☒	☐	Dual use research of concern

Methods

n/a	Involved in the study	
☒	☐	ChIP-seq
☐	☒	Flow cytometry
☒	☐	MRI-based neuroimaging

Antibodies

Antibodies used

- "Name" "Company" "Catalog Nr." "Lot Nr." "Dilution used" "Application"

Control-FITC, Miltenyi Biotec, 130-113-449, 5210504501, 1 to 50, Flow cytometry
SSEA4-FITC, Miltenyi Biotec, 130-122-918, 1322050485, 1 to 50, Flow cytometry
ACTN2-FITC, Miltenyi Biotec, 130-119-766, 1322011086, 1 to 50, Flow cytometry
Control-PE Miltenyi Biotec 130-113-762 5220607743 1 to 50 Flow cytometry
MLC2v-PE Miltenyi Biotec 130-119-680 1324020702 1 to 50 Flow cytometry
ACTN2, Abcam, AB9465, 1058926-5, 1 to 200, Immunoflourescence
Phalloidin, Invitrogen, A22287, 2326923, 1 to 400, Immunoflourescence
Wheat Germ Agglutinin, Invitrogen, W32466, 2126807, 1 to 400, Immunoflourescence
cTnT, Abcam, ab45932, GR3427142-1, 1 to 200, Immunoflourescence
Hoeschet, Life Technologies, H1399, 1924446, 1 to 500, Immunoflourescence
H2AFX, Cell signaling Technologies, 9718S, 17, 1 to 200, Immunoflourescence
Vimentin, R&D Systems, MAB2105, UUQ0222101, 1 to 200, Immunoflourescence
THY1, Abcam, ab133350, GR3368588-12, 1 to 200, Immunoflourescence
CD31 (PECAM1), Invitrogen, 17-0319-42, 2507946, 1 to 200, Immunoflourescence
Alexa 488 RB, Invitrogen, A32790, VC296619, 1 to 400, Immunoflourescence
Alexa 488 MS, Invitrogen, A21131, 1964395, 1 to 400, Immunoflourescence
Alexa 555 RB, Invitrogen, A31572, 2339822, 1 to 400, Immunoflourescence
Alexa 555 Rat, Invitrogen, A21434, 2329406, 1 to 800, Immunoflourescence
Alexa 555 MS, Invitrogen, A21045, 706307, 1 to 400, Immunoflourescence
cTnT, Life Technologies, MA512960, -, 1 to 800, Western Blot
GAPDH, Cell signaling Technologies, 2118S, -, 1 to 800, Western Blot

Validation

All antibodies were commercially available, validated and suitable for human species as specified by the manufacturer.

Eukaryotic cell lines

Policy information about cell lines and Sex and Gender in Research

Cell line source(s)

WTC-11 (Ctrl) is a wild-type human male iPSC line (Coriell Institute: # GM25256) that harbors a doxycycline (Dox)-inducible CRISPR/Cas9, which was created by introducing CAG-rtTA::TetO-Cas9 into the AAVS1 locus (Addgene #73500; Mandegar et al., 2016 Cell Stem Cell). Additional cell lines (RCM, Barth, Calmodulinopathy, ACM) were introduced into the Ctrl line using Cas9 genome editing, as described (Wang et al., 2017 Nature Protocols) Patient-derived lines (1, 2, 3 and 4) were reprogrammed from blood using the CytoTune™-iPS 2.0 Sendai Reprogramming Kit (Thermo Fisher Scientific, # A16517).

Authentication

Regular genotyping for the affected locus in iPSC lines was performed using PCR and Sanger sequencing.

Mycoplasma contamination

Mycoplasma testing was performed in iPSC of all used lines (LookOut® Mycoplasma PCR Detection Kit Optimized for use with JumpStart™ Taq DNA Polymerase, D9307; Sigma Aldrich # MP0035-1KT).

Commonly misidentified lines
(See ICLAC register)

No commonly misidentified lines were used in this study.

Flow Cytometry

Plots

Confirm that:

- ☐ The axis labels state the marker and fluorochrome used (e.g. CD4-FITC).
- ☐ The axis scales are clearly visible. Include numbers along axes only for bottom left plot of group (a 'group' is an analysis of identical markers).
- ☐ All plots are contour plots with outliers or pseudocolor plots.
- ☒ A numerical value for number of cells or percentage (with statistics) is provided.

Methodology

Sample preparation	FACS analysis was done for intracellular and extracellular antigens as previously described in detail by Breckwoldt et al. 2017 (Nature Protocols).
Instrument	BD Fortessa (BD Biosciences)
Software	BD FACSDiva (BD Biosciences)
Cell population abundance	1,000-10,000 events (cells) were measured for statistical evaluation of the cell population.
Gating strategy	Gating strategy was performed as published previously in Breckwoldt et al. 2017 (Nature Protocols).<br><br>Forward and side scatter were used to set up inital gating for the target population the starting cell population to isolate cardiac troponinT-FITC-positive or SSEA4-FITC-positive cells with the help of cells stained with REA Control (I)-FITC isotype control.

☐ Tick this box to confirm that a figure exemplifying the gating strategy is provided in the Supplementary Information.
